# Obstacles and Strategies in Nursing Care for Migrant, Asylum-Seeking, and Refugee Children: A Scoping Review

**DOI:** 10.1177/10436596261463223

**Published:** 2026-07-17

**Authors:** Carolina Marcelino, Tânia Marcelino, Sílvia Ramos, Joana Romeiro, Sílvia Caldeira

**Affiliations:** 1 Universidade Católica Portuguesa, Lisbon, Portugal; 2 Escola Superior de Saúde de Santarém, Portugal; 3 Escola Superior de Enfermagem S. Francisco das Misericórdias, Lisbon, Portugal; 4 Centre for Interdisciplinary Research in Health: Faculty of Health Sciences and Nursing, Lisbon, Portugal

**Keywords:** child, health, migration, obstacles, strategies

## Abstract

**Introduction:**

The migratory phenomenon is reaching record levels, with children as a vulnerable group, facing challenges during travel and adaptation. This review aims to map the obstacles and strategies in the provision of nursing care to migrant, asylum-seeking, and refugee children.

**Methods:**

This is a scoping review according to JBI guidelines; searches were conducted in June – July 2024 and updated in May 2026 in MEDLINE Complete, PubMed, CINAHL Complete, and Web of Science. Inclusion criteria were language (Portuguese, English, or Spanish), population (migrant children), and various settings.

**Results:**

Ten studies identified several obstacles to nursing care, including cultural barriers, communication difficulties, nurses’ attitudes, inadequate translation services, and limited knowledge of the health care system.

**Discussion:**

Culturally congruent nursing care is essential for improving outcomes for vulnerable children. This review emphasizes the need for inclusive health policies and ongoing training to promote cultural competence among nurses.

## Introduction

Globally, millions of children are affected by migration and forced displacement. Depending on the circumstances surrounding their displacement, these children may be classified as migrants, asylum seekers, or refugees (International Data Alliance for Children on the Move, 2023). While these categories carry distinct legal meanings, they often overlap in lived experience, particularly regarding instability, vulnerability, and barriers to accessing health care.

Migrant children are those who, whether accompanied by their parents or not, leave their country “not because of a direct threat of persecution or death, but mainly to better their lives . . .” (Alto Comissariado das Nações Unidas para os Refugiados, 2015). In contrast, asylum-seeking children are those who seek international protection but have either not yet applied for refugee status or are awaiting a decision on their application (United Nations High Commissioner for Refugees, 2006). According to the 1951 Convention Relating to the Status of Refugees, a refugee child is someone who, “fearing for good reason to be persecuted (. . .), finds themselves outside the country of which they are a national and cannot or, in virtue of that fear, does not wish to seek the protection of that country.” (Organização das Nações Unidas, 1967, p. 2).

Although legally distinct, these groups will hereafter be collectively referred to as displaced children, as they share common vulnerabilities associated with mobility, uncertainty of legal status, and structural barriers to protection and care. By the end of 2022, there were approximately 35.5 million migrant children, 1.5 million children seeking asylum, and 16 million refugee children worldwide (International Data Alliance for Children on the Move, 2023), highlighting the global magnitude of this phenomenon.

Displaced children may be exposed to potentially traumatic experiences before departure, during transit, and after arrival in host countries, including violence, armed conflict, family separation, exploitation, trafficking, and human rights violations. The aforementioned adversities can significantly affect their physical, cognitive, and emotional development and are associated with a higher prevalence of physical and mental health problems compared with other children in the host country, particularly in situations of prolonged displacement (Baauw et al., 2019; Blackmore et al., 2020; Höhne et al., 2023). These experiences may also increase their need for timely, accessible, and culturally sensitive health care services.

In response to these vulnerabilities, Article 24.º of the Convention on the Rights of the Child affirms that every child “(. . .) has the right to enjoy the highest attainable standard of health and to benefit from medical services (. . .).” It further emphasizes that, through international cooperation, States must ensure that no child is denied access to effective health care services (Fundo das Nações Unidas para a Infância [UNICEF], 2019, p. 20). Despite these internationally recognized rights, structural, legal, linguistic, cultural, and institutional barriers continue to limit displaced children’s access to adequate and equitable health care.

In this context, nursing is expected to play an essential role in promoting the health and well-being of displaced children. Nurses frequently act at the frontline of service delivery and may contribute through clinical care, care coordination, advocacy, cultural mediation, and family-centered support. Nonetheless, nurses’ capacity to fulfill this role may be constrained by systemic, organizational, and contextual challenges.

Although the health needs of displaced children have been widely documented, evidence specifically addressing the obstacles encountered by nurses in providing care to migrant, asylum-seeking, and refugee children remains scattered across disciplines and settings. Mapping this body of evidence is essential to clarify the scope of existing knowledge, identify gaps, and inform future research, practice, and policy.

## Methods

A Scoping Review was conducted according to the JBI guidelines (Peters et al., 2024), to answer the following search question: “What are the obstacles and strategies in the provision of nursing care to migrant, asylum-seeking, and refugee children?” aiming to map the obstacles and strategies involved in the provision of nursing care to migrant, asylum-seeking, and refugee children. This protocol has been registered in the Open Science Framework (available at https://doi.org/10.17605/OSF.IO/G5UEW).

According to the JBI guidelines, the inclusion criteria were based on the mnemonic “PCC,” which stands for participants, concept, and context. The review considered studies including migrant children, asylum-seeking children, and refugee children aged from 0 to 18 years. The concept of interest comprised barriers and/or enablers in the provision of nursing care. The context included any setting in which nursing care is delivered, such as schools, refugee camps, primary health care services, and hospitals. The search included all studies indexed in each database from inception until July 2024, and updated in May 2026. Studies published in English, Portuguese, and Spanish were considered, as these are the languages spoken by the review team.

A broad range of study designs was considered eligible for inclusion, including qualitative, quantitative, and mixed-methods studies, as well as other relevant evidence sources addressing the research question.

The search strategy aimed to find published studies and was organized into three stages. In June 2024, a preliminary search in CINAHL Complete and PubMed was conducted to identify search terms and verify the feasibility of the topic. In July 2024, the second phase of the research was conducted with the collaboration of a scientific librarian, aiming at the definition of search terms and the construction of the complete search strategy adapted to the different databases (MEDLINE Complete, PubMed, CINAHL Complete, Web of Science) and gray literature (*Repositório Científico de Acesso Aberto de Portugal*) and updated in May 2026. The search strategy is described in Table 1.

**Table 1.** Search Strategy.

Base date	Search strategy	Results
CINAHL Complete EBSCO Host (18/05/2026)	(XB (child* OR infant* OR adolescent* OR teen* OR newborn*) OR MH (Child OR Infant OR adolescence OR “Infant, Newborn”)) AND (XB (migration OR migrant* OR refugee* OR “asylum seeker*” OR Transient* OR “Displaced Person*”) OR MH (Migrants OR Refugees)) AND (XB (barrier* OR obstacle* OR challenge* OR difficult* OR issue* OR problem* OR strateg* OR facilitator*)) AND (XB (nurs* OR “nursing care”) OR (MH “nursing care”))	273
*Medline Complete EBSCO Host* (18/05/2026)	(XB (child* OR infant* OR adolescent* OR teen* OR newborn*) OR MH (Child OR Infant OR adolescent OR “Infant, Newborn”)) AND (XB (migration OR migrant* OR refugee* OR “asylum seeker*” OR Transient* OR “Displaced Person*”) OR MH (“Transients and Migrants” OR refugees)) AND (XB (barrier* OR obstacle* OR challenge* OR difficult* OR issue* OR problem* OR strateg* OR facilitator*)) AND (XB (nurs* OR “nursing care”) OR MH (“nursing care”))	361
*PubMed* (18/05/2026)	((((((((child*[Title/Abstract] OR infant*[Title/Abstract] OR adolescent*[Title/Abstract] OR teen*[Title/Abstract] OR newborn*[Title/Abstract]) OR (Child[MeSH Terms])) OR (Child[MeSH Terms])) OR (Infant[MeSH Terms])) OR (adolescent[MeSH Terms])) OR (“Infant, Newborn”[MeSH Terms])) AND (((migration[Title/Abstract] OR migrant*[Title/Abstract] OR refugee*[Title/Abstract] OR “asylum seeker*”[Title/Abstract] OR Transient*[Title/Abstract] OR “Displaced Person*”[Title/Abstract]) OR (“Transients and Migrants”[MeSH Terms])) OR (refugees[MeSH Terms]))) AND (barrier*[Title/Abstract] OR obstacle*[Title/Abstract] OR challenge*[Title/Abstract] OR difficult*[Title/Abstract] OR issue*[Title/Abstract] OR problem*[Title/Abstract] OR strateg*[Title/Abstract] OR facilitator*[Title/Abstract])) AND ((nurs*[Title/Abstract] OR “nursing care”[Title/Abstract]) OR (“nursing care”[MeSH Terms]))	432
*Web of Science Core Collection* (18/05/2026)	TS=(child* OR infant* OR adolescent* OR teen* OR newborn*) AND TS=(migration OR migrant* OR refugee* OR “asylum seeker*” OR Transient* OR “Displaced Person*”) AND TS=(barrier* OR obstacle* OR challenge* OR difficult* OR issue* OR problem* OR strateg* OR facilitator*) AND TS=(nurs* OR “nursing care”)	379
*RCAAP via B-ON* (18/05/2026)	((TI (child* OR infant* OR adolescent* OR teen* OR newborn*)) OR (AB (child* OR infant* OR adolescent* OR teen* OR newborn*)) OR (SU (child* OR infant* OR adolescent* OR teen* OR newborn*))) AND ((TI ((migration OR AND migrant*) OR refugee* OR “asylum seeker*” OR Transient* OR “Displaced Person*”)) OR (AB ((migration OR AND migrant*) OR refugee* OR “asylum seeker*” OR Transient* OR “Displaced Person*”)) OR (SU ((migration OR AND migrant*) OR refugee* OR “asylum seeker*” OR Transient* OR “Displaced Person*”))) AND ((TI (barrier* OR obstacle* OR challenge* OR difficult* OR issue* OR problem* OR strateg* OR facilitator*)) OR (AB (barrier* OR obstacle* OR challenge* OR difficult* OR issue* OR problem* OR strateg* OR facilitator*)) OR (SU (barrier* OR obstacle* OR challenge* OR difficult* OR issue* OR problem* OR strateg* OR facilitator*))) AND ((TI (nurs* OR “nursing care”)) OR (AB (nurs* OR “nursing care”)) OR (SU (nurs* OR “nursing care”))) Filter by: Content provider: RCAAP	8

Through the applied search strategy, a total of 1,453 articles were identified in English, Portuguese, and Spanish. All records were compiled and imported into the Zotero reference management software, where duplicates were removed. The remaining records were then uploaded to the Rayyan Intelligent Systematic Review platform for screening. Two independent reviewers conducted the initial screening based on titles and abstracts, followed by full-text review in accordance with the established inclusion criteria. Any discrepancies between reviewers throughout the process were resolved through discussion and consensus. A total of 10 studies met the inclusion criteria and were included in the final review. In addition, the reference lists of included studies were screened to identify further relevant publications. The study selection process is presented in Figure 1.

**Figure 1.**
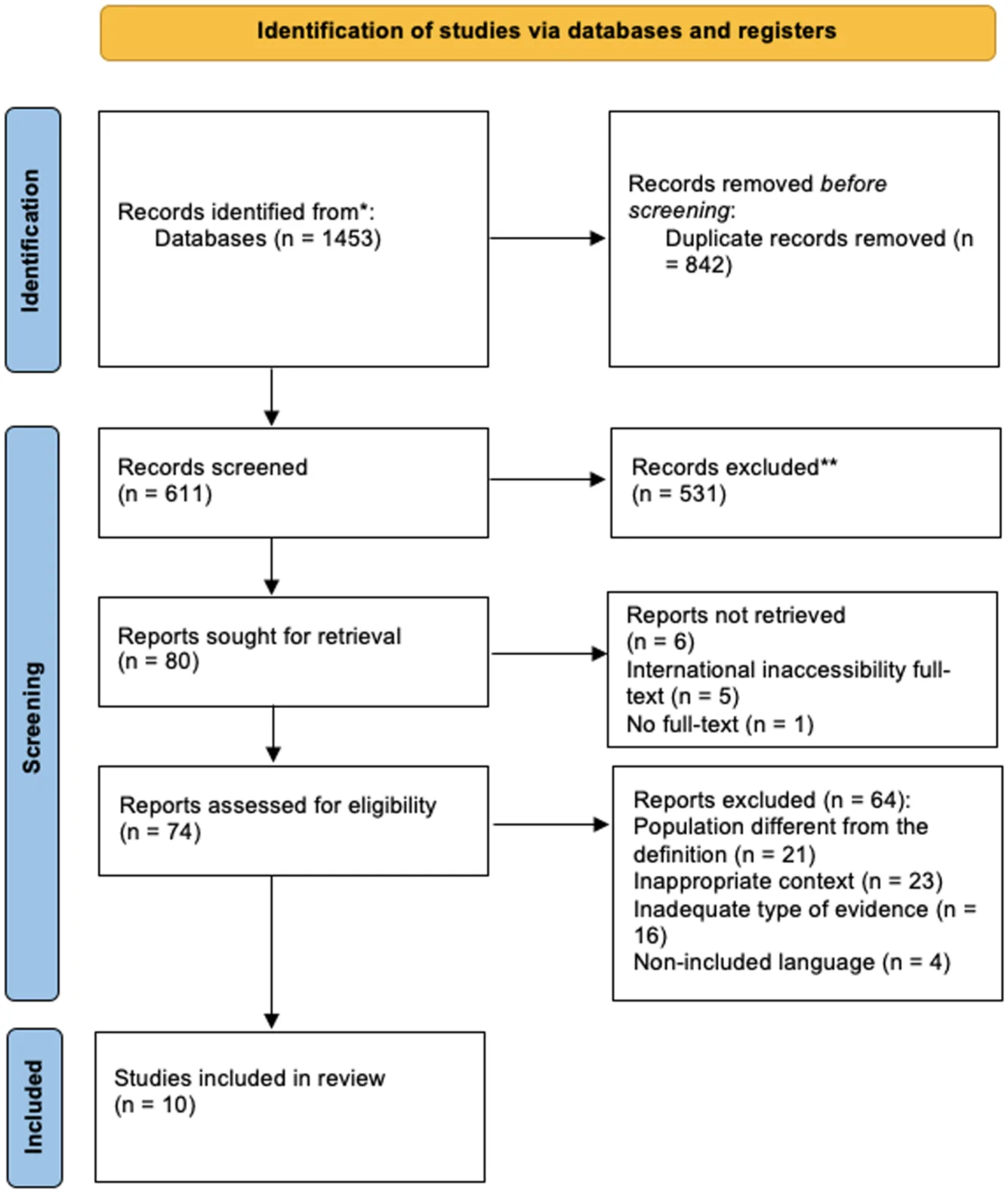
PRISMA-ScR = Preferred Reporting Items for Systematic reviews and Meta-Analyses extension for Scoping Reviews.

The articles were analyzed, and categories for organizing the results were created based on primary-level obstacles and strategies. Within this framework, content analysis was conducted to ensure the specificity and exclusivity of each category in each dimension, among the review team.

## Data Analysis and Presentation

Interestingly, of the 10 studies that met the inclusion criteria, all were qualitative. These studies used phenomenological, ethnographic, and other qualitative approaches to examine the experiences and perceptions of nurses, parents, and families.

The studies included in this analysis were published between 2012 and 2023 and involved nurses providing care to migrant children, asylum seekers, and refugees, as well as the parents of these children. Across the included studies, 208 participants were included. The characteristics of the included studies are summarized in Table 2.

**Table 2.** Characteristics of the Analyzed Studies.

Authors	Title	Source	Objective	Sample	Methodology	Results
Riggs et al. (2012)	Accessing maternal and Child health services in Melbourne, Australia: Reflections from refugee families and service providers	Australia	Explore the utilization and experience of maternal and child health services in Melbourne, Victoria, for parents of refugee background from the perspective of users and providers	87 mothers from Karen, Iraqi, Assyrian Chaldean, Lebanese, South Sudanese and Bhutanese backgrounds, who had lived an average of 4.7 years in Australia	Qualitative study	**Obstacles:** Lack of capacity of nurses to identify and meet the needs of children and refugee parents. Language barrier. Lack of time for consultation. Lack of access to translated information. Nurses lack knowledge of children’s rights. Refugees have a lack of knowledge and support about the Australian health system. **Strategies:** Facilitate access to maternal health services, child health, home visits and facilitation in scheduling appointments. Building a trusting relationship through continuous involvement between the nurse, the translator and the family. In-person translators. Training of 0nurses in cultural competence.
Tavallali et al. (2017)	Cross-cultural care encounters in pediatric care: minority ethnic parents’ experiences.	Sweden	Describe the expectations and experiences of cross-cultural care encounters among minority ethnic parents in Swedish pediatric care.	12 parents of minority ethnic	Qualitative Study	**Obstacles:** Language barrier between nurses, the children and their families, which hinders the establishment of a trusting relationship. Lack of familiarity with the culture of the host country. **Strategies:** Establishing a trusting and respectful relationship between the nurses and the families. Training of nurses in competence and cultural sensitivity. Respect for religious beliefs.
Tulli et al. (2020)	Immigrant Mothers’ Perspectives of Barriers and Facilitators in Accessing Mental Health Care for Their Children	Canada	Explore immigrant and refugee mothers’ perceptions of barriers and facilitators for mental health care for their children in Edmonton, Alberta, Canada.	18 mothers, immigrant and refugee	Descriptive, Qualitative Study	**Obstacles**: Financial difficulties, lack of knowledge and support about the Canadian health system. Language barrier, racism’s perception and discrimination in health services. Excessive bureaucracy in accessing health care. **Strategies:** Increase in the number of accessible translators with training in scientific language. Support from the school nurse in accessing and coordinating with health services. An increase in parents’ health literacy allows them to navigate health services. Culturally congruent and family-centered care. Training of nurses in cultural competence.
Kaufmann et al. (2020)	Communication challenges between nurses and migrant pediatric patients	Switzerland	Examine which communicative challenges hospital nurses are confronted with in the care of migrant pediatric patients and how they cope with them. Examine what requirements nurses (and other stakeholders) have regarding a digital communication aid to improve the care of migrant pediatric patients in the hospital setting.	9 pediatric hospital nurses	Qualitative Study	**Obstacles:** Language barrier, cultural barrier, and lack of health literacy (poor adherence or inadequate management of the therapeutic plan). Migrant population with precarious working conditions. Increased time in providing care due to the linguistic and cultural barrier. Lack of a health history of the child. Difficulty in obtaining informed consent and developing a care plan. Lack of a standardized and systematic procedure to deal with the language barrier. **Strategies:** Establish a trusting relationship with the child and family. Use of a qualified translator. Use of religion and prayer to support religious beliefs. Training of nurses in cultural competence.
Barghadouch et al. (2020)	The Care Ethics of Child Health Nurses in Danish Asylum Centers: An Ethnographic Study	Denmark	Examine the ethical care practices that child health nurses within Danish asylum centers adopt to overcome barriers related to culture, language and migration history, in delivering care.	20 families and 6 child health nurses	Ethnographic Study	**Obstacles:** Legislative and structural obstacles. Lack of child health history. **Strategies:** Involvement and empathy of nurses, allowing the creation of a trusting relationship between the nurse and families. Explanation about the neutrality of nurses, emphasizing the separation of their role in relation to immigration and police authorities. Flexibility and adaptation of care to each child and family. Respect for the traditions of families, as long as they do not jeopardize the health and well-being of children.
Tek et al. (2021)	Challenges Experienced by Nurses Caring for Syrian Refugee Children	Turkey	Determine the challenges experienced by the nurses providing care to Syrian pediatric patients.	13 nurses are working in the pediatric service of a hospital that provides care for Syrian pediatric patients.	Qualitative Study	**Obstacles:** Language barrier between nurses and the children and their families. Cultural differences regarding nutrition and hygiene care. Insufficient number of translators and a lack of training in scientific language. The financial and bureaucratic problems have a negative impact on access to nursing care. Increased time of provision of care due to the language and cultural barrier. **Strategies:** Accessible translators in the wards with training in scientific language. Training of nurses in competence and cultural sensitivity. Empathy of the nurses for the situations experienced by refugees.
Kvamme & Voldner (2022)	Public health nurses’ encounters with undocumented migrant mothers and children	Norway	Describe how the public health nurses experienced challenges and dilemmas in ensuring the best interests of the undocumented migrant child.	7 public health nurses	Descriptive, Qualitative Study	**Obstacles:** Nurses lack knowledge of the rights of undocumented migrant children. **Strategies:** Building a trusting relationship with the child and family through increased time in providing care. Clarification on the neutrality of nurses, emphasizing the separation of their role in relation to the authorities of immigration.
Öner et al. (2023)	Challenges, expectations, and cultural care experiences of nurses regarding migrant children receiving burn treatment and their caregivers: A qualitative study	Turkey	Reveal the challenges, expectations, and cultural care experiences of nurses regarding migrant children receiving burn treatment and their caregivers.	12 nurses	Descriptive Qualitative Study	**Obstacles:** Language barrier between nurses, the children and their families, hindering the establishment of trust and contributing to the increase in time in the provision of care. Cultural and religious differences. **Strategies:** Accessible translators in the wards with training in scientific language. Access to information translated into the native language. Training of nurses in cultural competence.
Top (2023)	The Challenges in the Care of Immigrant Children in the Clinic: A Phenomenological Study	Turkey	Examine the challenges experienced by pediatric nurses providing care for the children of immigrant families and contribute to the solutions that can be provided.	16 nurses	Phenomenological Study	**Obstacles:** Language barrier between nurses and the children and their families. Insufficient number of translators. Cultural differences that lead to adaptation problems in the hospital context. Difficulty in explaining interventions and, consequently, difficulty in obtaining informed consent. Increased time of care due to the language and cultural barrier. Low level of education of migrant parents. Cultural differences regarding hygiene care. **Strategies:** Increase the number of translators and access to translated information in the native language. Training of nurses in competence and cultural sensitivity.
Inkeroinen et al. (2023)	School Nurses’ Experiences of Health Promotion for School-Age Asylum Seekers	Finland	Describe school nurses’ experiences of providing health promotion to school-age asylum seekers.	12 nurses of the health school (public health)	Descriptive, Qualitative Study	**Obstacles:** Cultural differences and language barrier. Nurses lack training in mental health and lack of knowledge of the rights of asylum-seeking children. Increased time in providing care due to the linguistic and cultural barrier. Lack of a health history of the child. **Strategies:** Existence of a school nurse who promotes care. partnership with the child and family. Accessible translators trained in scientific language. Training of nurses in cultural competence.

### Obstacles to Nursing Care for Migrant, Asylum Seekers, and Refugee Children

The obstacles experienced by nurses and families in the provision of nursing care to migrant children, asylum seekers, and refugee children included cultural and linguistic barriers, increased nursing workload, limited knowledge among nurses regarding migrants’ rights, racism and discrimination, and bureaucratic constraints. Families also faced difficulties related to limited knowledge of the health care system, financial difficulties, low educational levels, lack of translated information, and the insufficient number of interpreters. These barriers are summarized in Table 3.

**Table 3.** Obstacles in Nursing Care.

Authors	Cultural barrier	Linguistic barrier	Increase in nurses’ workload	Nurses lacking knowledge of rights	Racism/ discrimination	Bureaucracy	Lack of knowledge of the healthcare system	Financial difficulties	Level of education	Lack of information translated	Insufficient number of interpreters
Riggs et al. (2012)	**X**	**X**	**X**	**X**			**X**		**X**	**X**	
Tavallali et al. (2017)	**X**	**X**									
Tulli et al. (2020)	**X**	**X**			**X**	**X**	**X**	**X**	**X**		
Kaufmann et al. (2020)	**X**	**X**	**X**					**X**			
Barghadouch et al. (2020)						**X**					
Tek et al. (2021)	**X**	**X**	**X**					**X**			**X**
Kvamme & Voldner (2022)				**X**							
Öner et al. (2023)	**X**	**X**	**X**								
Top (2023)	**X**	**X**	**X**						**X**		**X**
Inkeroinen et al. (2023)	**X**	**X**	**X**	**X**							

Linguistic and cultural barriers were identified as significant challenges in most of the studies included. Difficulties related to language differences and cultural misunderstandings were reported by both nurses and parents, hindering effective communication and the development of therapeutic relationships. These barriers were reported to increase nurses` workload, as additional time is required to negotiate care plans, obtain informed consent, and understand the cultural context of each family (Inkeroinen et al., 2023; Kaufmann et al., 2020; Öner et al., 2023; Riggs et al., 2012; Tek et al., 2021; Top, 2023).

Another major obstacle identified in several studies was the lack of accessible health histories for migrant, asylum-seeking, and refugee children (Barghadouch et al., 2020; Inkeroinen et al., 2023; Kaufmann et al., 2020; Riggs et al., 2012). This issue is often compounded by language barriers and the loss of medical documentation during migration or displacement. As a result, health care professionals frequently lack essential information regarding vaccination status, chronic conditions, and previous treatments when these children arrive in host countries.

Limited knowledge among nurses regarding the human rights and health rights of migrant and refugee children was also identified as a barrier to the delivery of appropriate care (Inkeroinen et al., 2023; Kvamme & Voldner, 2022; Riggs et al., 2012). This lack of knowledge may hinder nurses’ ability to advocate effectively for this vulnerable population within health care systems.

Considered from parents’ perspective, several studies reported feelings of insufficient support upon arrival in the host country (Riggs et al., 2012; Tulli et al., 2020). These experiences were largely associated with unfamiliarity with the organization and functioning of the host country’s health care system, including available services and access procedures.

Furthermore, legislative, structural, and bureaucratic barriers were identified as additional challenges that may delay access to child health services (Barghadouch et al., 2020; Tulli et al., 2020). Some parents also reported experiences of discrimination from health care professionals, which negatively influenced their willingness to seek health care for their children.

Socioeconomic factors were also reported as relevant obstacles. Financial hardship among migrant, asylum-seeking, and refugee families limits the ability to obtain necessary health care resources (Kaufmann et al., 2020; Tek et al., 2021; Tulli et al., 2020). In addition, low educational attainment among many parents was associated with reduced health literacy, which may affect their understanding of health information and adherence to treatment recommendations (Riggs et al., 2012; Top, 2023; Tulli et al., 2020).

Finally, the lack of communication tools tailored to this population was identified as a significant barrier to effective care. These challenges include the lack of informational materials in the families’ native languages, a shortage of qualified interpreters, and limited expertise in translating complex scientific or medical terminology (Riggs et al., 2012; Tek et al., 2021; Top, 2023). These factors may undermine communication and hinder the establishment of trust between nurses, children, and their families.

### Strategies in Nursing Care for Migrant, Asylum Seekers, and Refugee Children

A range of strategies to facilitate nursing care for migrant, asylum-seeking, and refugee children was identified across the selected studies. These included the presence of a school nurse, training nurses and other health care professionals in cultural competence, the use of interpreters, and training on health services and access pathways. Other relevant strategies included establishing a relationship of trust with children and families, respecting beliefs and traditions, allocating extra time for care, and promoting cultural sensitivity and nurses’ neutrality in nursing practice. Furthermore, the provision of translated information in families’ native languages was identified as an important measure to improve communication and health literacy. These strategies are summarized in Table 4.

**Table 4.** Facilitating Strategies in Nursing Care.

Authors	School Nurse	Training nurses in cultural competences	Interpreters	Training on health services	Relationship of trust	Respect beliefs/traditions	Extra time for care	Translated Information	Neutrality of the nurse	Cultural sensitivity
Riggs et al. (2012)		**X**	**X**		**X**					
Tavallali et al. (2017)		**X**			**X**	**X**				**X**
Tulli et al. (2020)	**X**	**X**	**X**	**X**						**X**
Kaufmann et al. (2020)		**X**	**X**		**X**	**X**				**X**
Barghadouch et al. (2020)					**X**	**X**			**X**	**X**
Tek et al. (2021)		**X**	**X**							**X**
Kvamme & Voldner (2022)					**X**		**X**		**X**	
Öner et al. (2023)		**X**	**X**					**X**		
Top (2023)		**X**	**X**					**X**		
Inkeroinen et al. (2023)	**X**	**X**	**X**	**X**			**X**			

Several studies highlighted the importance of establishing a strong partnership between nurses, children, and their families as a foundation for effective care (Barghadouch et al., 2020; Kaufmann et al., 2020; Kvamme & Voldner, 2022; Riggs et al., 2012; Tavallali et al., 2017). Building trust was identified as a key element in this process.

To foster such partnerships, nurses must develop cultural competence and demonstrate respect for the beliefs, values, and traditions of migrant and refugee populations (Barghadouch et al., 2020; Inkeroinen et al., 2023; Kaufmann et al., 2020; Öner et al., 2023; Riggs et al., 2012; Tavallali et al., 2017; Tek et al., 2021; Top, 2023; Tulli et al., 2020). Cultural sensitivity in communication and care practices was also emphasized as an essential component of high-quality nursing care (Barghadouch et al., 2020; Riggs et al., 2012; Tavallali et al., 2017; Tek et al., 2021; Tulli et al., 2020).

In the case of undocumented migrants, it is particularly important for nurses to clarify the separation between health care services and immigration or political authorities. This reassurance may help reduce fear among families and promote greater engagement with health care services, reinforcing the ethical commitment of nurses to human rights and professional neutrality (Barghadouch et al., 2020; Kvamme & Voldner, 2022). In addition, dedicating more time to interactions with migrant families was identified as a strategy that fosters the development of trust and enables nurses to better understand the specific needs of each family (Inkeroinen et al., 2023; Kvamme & Voldner, 2022).

School nurses were also identified as important facilitators of health care access. By maintaining close relationships with children and their families within the school environment, they can support families’ adaptation to the host country and help connect them with relevant health care services (Inkeroinen et al., 2023; Tulli et al., 2020).

Communication strategies were widely emphasized across the included studies. The use of trained interpreters was considered essential for bridging communication gaps between health care professionals and migrant families (Inkeroinen et al., 2023; Kaufmann et al., 2020; Öner et al., 2023; Riggs et al., 2012; Tek et al., 2021; Top, 2023; Tulli et al., 2020). However, interpreters must possess adequate proficiency in scientific terminology to ensure accurate and effective communication.

Finally, the development and dissemination of educational materials in the native languages of migrant children and their families were identified as additional strategies to enhance understanding and promote health literacy (Öner et al., 2023; Top, 2023). These resources helped reduce communication barriers, improve families’ understanding of health information, and facilitate access to and navigation of the health care system.

## Discussion

This scoping review aimed to map the main barriers and facilitating strategies in nursing care for migrant, asylum-seeking, and refugee children and their families. The findings indicate that communication barriers, structural challenges within host-country health care systems, socioeconomic vulnerability, and cultural differences significantly influence the accessibility and quality of nursing care. At the same time, the review highlights several strategies that may support more effective care, including culturally competent nursing practice, the use of interpreters, trust-building with families, and the involvement of school nurses in supporting migrant children.

### Structural Barriers Within the Host-Country Health System

The findings of this review suggest that migrant and refugee families frequently encounter structural and organizational barriers when attempting to access health care services. Difficulties navigating administrative procedures, legal restrictions, and limited knowledge of the health care system organization were identified as important obstacles to care (Barghadouch et al., 2020; Riggs et al., 2012; Tulli et al., 2020). These challenges may delay access to health care and contribute to inequalities in health outcomes among migrant children.

These findings are consistent with previous research indicating that health care systems are often insufficiently prepared to meet the needs of increasingly diverse populations (Durbeej et al., 2021; Pereira Ramos, 2021). Migration processes frequently involve disruptions in access to health care and difficulties in continuity of care, particularly when medical documentation is unavailable or incomplete, as identified in some of the studies included in this review (Barghadouch et al., 2020; Inkeroinen et al., 2023; Kaufmann et al., 2020; Riggs et al., 2012). Strengthening health system responses through inclusive policies and improved coordination between health and social services may therefore be essential to ensuring equitable access to care for migrant children.

These issues are closely aligned with the United Nations Sustainable Development Goal 3, which emphasizes the importance of ensuring healthy lives and promoting well-being for all individuals, regardless of their social or legal status (Organização das Nações Unidas, 2024). In this context, health care systems must develop policies and organizational frameworks that promote equitable and culturally responsive health care delivery (Willey et al., 2022).

### Communication and Culturally Responsive Care

Communication challenges emerged as one of the most significant barriers in the provision of nursing care for migrant children and their families. Language differences and cultural misunderstandings may limit effective communication between nurses and families, hinder the development of therapeutic relationships, and increase the risk of misinterpretation of health information. Several studies included in this review highlighted language barriers as a major challenge in nursing practice (Inkeroinen et al., 2023; Kaufmann et al., 2020; Öner et al., 2023; Riggs et al., 2012; Tek et al., 2021; Top, 2023; Tulli et al., 2020). The use of trained interpreters was frequently identified as a key strategy for facilitating communication and ensuring that families are able to understand health information and participate in care decisions (Inkeroinen et al., 2023; Kaufmann et al., 2020; Öner et al., 2023; Riggs et al., 2012; Tek et al., 2021; Top, 2023; Tulli et al., 2020). However, some studies also reported limitations in the availability of qualified interpreters and in their ability to accurately translate complex medical terminology (Tek et al., 2021; Top, 2023). Previous research similarly emphasizes the importance of interpreter services as a fundamental component of safe and equitable health care for migrant populations (Willey et al., 2022).

In addition to interpreter services, providing health information in migrant families’ native languages may significantly improve understanding and promote informed decision-making. Some studies included in this review highlighted the lack of translated educational materials as a barrier to effective communication and individualized care planning (Öner et al., 2023; Riggs et al., 2012; Top, 2023). Developing culturally and linguistically appropriate communication strategies may therefore represent an important step toward improving health care access and treatment adherence among migrant families (Pereira Ramos, 2021; Willey et al., 2022).

### Socioeconomic Vulnerability and Health Inequalities

This review also highlights the influence of socioeconomic vulnerability on health care access and experiences among migrant and refugee families. Forced migration is frequently associated with financial instability, precarious employment, and limited educational opportunities, factors that may reduce families’ capacity to access health care resources and navigate complex health systems (Pereira Ramos, 2021). Several studies included in this review reported that low levels of parental education and limited health literacy can hinder families’ understanding of health information and complicate the implementation of nursing interventions (Riggs et al., 2012; Top, 2023; Tulli et al., 2020).

Financial difficulties may also affect families’ ability to acquire essential resources needed for their children’s care, even when health care services are available (Kaufmann et al., 2020; Tek et al., 2021; Tulli et al., 2020). These structural determinants of health contribute to persistent health inequalities among migrant populations and highlight the need for integrated responses that address both health care and social support needs.

In addition, experiences of discrimination within health care settings were reported in some studies (Tulli et al., 2020), suggesting that migrant families may encounter barriers not only at structural levels but also within interpersonal interactions. Health care professionals, therefore, have a critical responsibility to ensure equitable and respectful care. According to the International Council of Nurses, nurses must protect and advocate for the health rights of all individuals, acting as agents of change to promote inclusion and reduce inequalities in health care access (International Council of Nurses, 2011; Truong et al., 2021).

### The Role of Culturally Competent Nursing Care

Culturally competent nursing care emerged as a key strategy for improving health care experiences among migrant children and their families. Cultural competence involves recognizing and respecting diverse values, beliefs, and health practices while adapting care to meet the specific needs of culturally diverse populations. Several studies included in this review highlighted the importance of cultural sensitivity in nursing interactions and its role in establishing trust between nurses and families (Barghadouch et al., 2020; Riggs et al., 2012; Tavallali et al., 2017; Tek et al., 2021; Tulli et al., 2020).

Leininger’s theory of transcultural nursing emphasizes the importance of culturally congruent care in promoting health and well-being among diverse populations (Leininger, 1988). In multicultural health care environments, nurses must develop cultural awareness and avoid ethnocentric assumptions to deliver respectful and individualized care (George, 2014).

Trust-building was also identified as a fundamental element of effective nursing care. Studies included in this review reported that when nurses demonstrate respect for cultural beliefs and provide empathetic communication, families are more likely to trust health care professionals and engage with health care services (Barghadouch et al., 2020; Kaufmann et al., 2020; Kvamme & Voldner, 2022; Riggs et al., 2012; Tavallali et al., 2017). This aspect is particularly relevant for undocumented migrants, who may fear contact with institutions due to concerns about immigration enforcement. In such cases, clearly distinguishing health care services from immigration authorities may help foster trust and encourage families to seek care when needed (Barghadouch et al., 2020; Kvamme & Voldner, 2022).

Trust-building was also identified as a fundamental element of effective nursing care. Studies included in this review reported that when nurses demonstrate respect for cultural beliefs and provide empathetic communication, families are more likely to trust health care professionals and engage with health care services (Barghadouch et al., 2020; Kaufmann et al., 2020; Kvamme & Voldner, 2022; Riggs et al., 2012; Tavallali et al., 2017). This aspect is particularly relevant for undocumented migrants, who may fear contact with institutions due to concerns about immigration enforcement. In such cases, clearly distinguishing health care services from immigration authorities may help foster trust and encourage families to seek care when needed (Barghadouch et al., 2020; Kvamme & Voldner, 2022).

### School Nurses as Facilitators of Health Care Access

Schools may represent important settings for promoting health and supporting the integration of migrant children and their families. The school environment provides opportunities for continuous contact between health care professionals, children, and families, allowing early identification of health needs and facilitating access to health care services. Some studies included in this review highlighted the role of school nurses in supporting migrant children’s adaptation to new environments and improving families’ access to health care services (Inkeroinen et al., 2023; Tulli et al., 2020).

Previous research similarly identifies schools as key community settings where social networks can be strengthened, and health information can be disseminated in a supportive and non-stigmatizing environment Durbeej et al. (2021). Strengthening collaboration between health care and educational institutions may therefore represent an effective strategy to improve health access and outcomes for migrant children.

## Implications for Nursing Practice and Policy

The findings of this review suggest several implications for nursing practice and health policy. For nursing practice, there is a clear need to strengthen training in cultural competence, migrant health, and communication strategies for working with linguistically diverse populations. Educational programs addressing migrant health rights and culturally responsive care may enhance nurses’ capacity to support migrant families and reduce barriers to health care.

At the policy level, health care systems should prioritize inclusive policies that ensure equitable access to pediatric health services regardless of migration status. Investment in interpreter services, culturally adapted health education materials, and integrated care pathways between health care and social support services may improve health care delivery for migrant populations.

## Implications for Future Research

Future research should explore the effectiveness of culturally adapted nursing interventions designed to improve communication, trust, and health outcomes among migrant children and their families. In addition, further studies are needed to examine the role of school-based health services in supporting migrant populations and to identify best practices for integrating health care, education, and social support systems in migrant health care.

## Limitations

Limitations include the significant heterogeneity of migrant populations, asylum seekers, and refugees, as well as the diversity of health care contexts in which nursing care is delivered, which complicates the identification of generalizable patterns. In addition, the review was limited to articles published in Portuguese, English, and Spanish, potentially excluding relevant studies available in other languages.

## Conclusion

Given the increasing mobility of populations worldwide, societies are becoming increasingly multicultural. This evolving reality demands that countries, societies, and individuals be prepared to embrace and navigate increasing cultural diversity. Nurses, in particular, must recognize the urgent need to enhance their cultural competencies to ensure the delivery of high-quality, culturally sensitive care for diverse and vulnerable populations.

This scoping review aimed to map the obstacles and strategies involved in providing nursing care to migrant children, asylum seekers, and refugees. Understanding the main challenges faced by both caregivers and the populations they serve is essential to improving health care access and outcomes for these children. Equally important is identifying effective solutions and strategies to address the needs of this vulnerable group while respecting their dignity and fundamental rights.

The scoping review identified several key obstacles, including cultural differences, communication barriers, nurse attitudes, the quality of translated materials, and an insufficient number of qualified interpreters. Other significant challenges included limited knowledge about the host country’s health system, low socioeconomic status, and low educational levels among most migrants, asylum seekers, and refugees, which may further increase their vulnerability and limit access to health care services.

As facilitating strategies, continuous nurse training is crucial to developing cultural competence and allows more time for building trusting relationships with these populations. Furthermore, the implementation of inclusive health policies is vital to guarantee equality, equity, and access to quality, culturally appropriate health care for migrant children, asylum seekers, and refugees. Providing care to these children requires sensitivity, empathy, and a sustained effort to overcome cultural and linguistic barriers. Only by doing so can health care meet their physical, psychological, and emotional needs, helping them integrate into new environments safely and with dignity while reducing health inequalities and promoting equitable care.

## Data Availability Statement

The data used in this study are available from the corresponding author upon reasonable request.
